# Federated Learning in Smart Healthcare: A Comprehensive Review on Privacy, Security, and Predictive Analytics with IoT Integration

**DOI:** 10.3390/healthcare12242587

**Published:** 2024-12-22

**Authors:** Syed Raza Abbas, Zeeshan Abbas, Arifa Zahir, Seung Won Lee

**Affiliations:** 1 Department of Bioscience, COMSATS University, Islamabad 45550, Pakistan; 2 Department of Precision Medicine, Sungkyunkwan University School of Medicine, Suwon 16419, Republic of Korea; zabbas@jbnu.ac.kr; 3 Department of Artificial Intelligence, Sungkyunkwan University, Suwon 16419, Republic of Korea; 4 Department of Metabiohealth, Sungkyunkwan University, Suwon 16419, Republic of Korea; 5 Personalized Cancer Immunotherapy Research Center, Sungkyunkwan University School of Medicine, Suwon 16419, Republic of Korea

**Keywords:** artificial intelligence, Internet of Things, machine learning, deep learning, healthcare, big data

## Abstract

Federated learning (FL) is revolutionizing healthcare by enabling collaborative machine learning across institutions while preserving patient privacy and meeting regulatory standards. This review delves into FL’s applications within smart health systems, particularly its integration with IoT devices, wearables, and remote monitoring, which empower real-time, decentralized data processing for predictive analytics and personalized care. It addresses key challenges, including security risks like adversarial attacks, data poisoning, and model inversion. Additionally, it covers issues related to data heterogeneity, scalability, and system interoperability. Alongside these, the review highlights emerging privacy-preserving solutions, such as differential privacy and secure multiparty computation, as critical to overcoming FL’s limitations. Successfully addressing these hurdles is essential for enhancing FL’s efficiency, accuracy, and broader adoption in healthcare. Ultimately, FL offers transformative potential for secure, data-driven healthcare systems, promising improved patient outcomes, operational efficiency, and data sovereignty across the healthcare ecosystem.

## 1. Introduction

### 1.1. Background and Motivation

The healthcare sector has seen extraordinary advancements in data-driven approaches, prompted largely by machine learning (ML) and deep learning (DL) applications that include personalized treatment plans, predictive analytics, and intelligent diagnostics [1]. However, healthcare data, because of its sensitive and private nature, offers unique challenges around privacy, security, and ethical data utilization. Traditional centralized ML methods need data to be compiled at a central location, which can increase privacy risks and conflict with healthcare protocols like the Health Insurance Portability and Accountability Act (HIPAA) and General Data Protection Regulation (GDPR). In reaction to these challenges, federated learning (FL) has become evident as a promising solution for maintaining data security without sacrificing the capability of ML and DL models.

**Figure 1:**
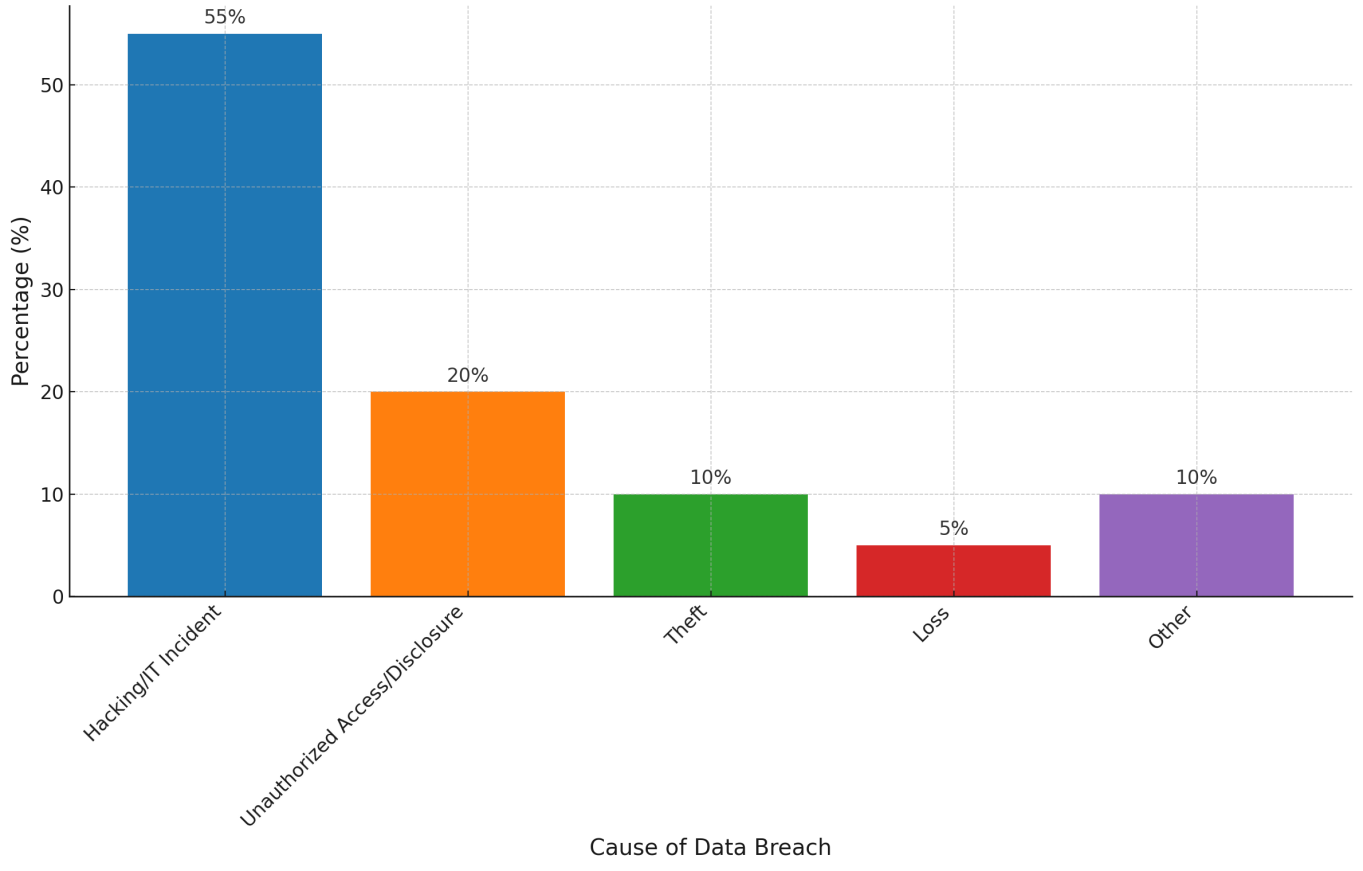
Percentage of healthcare data breaches by cause.

FL facilitates decentralized model training precisely at the database, which can involve mobile devices, hospitals, or any IoT-enabled health devices. This distributed strategy permits healthcare institutions to collectively take advantage of large-scale ML without sharing raw patient data, thus preserving privacy and agreements with data protection protocols [2]. By maintaining data within organizational limits and aggregating only model updates, FL reduces the risks related to data exposure and mishandling, making it a beneficial technique for modern smart health systems. Figure 1 shows the growing challenges in healthcare data security, emphasizing the role of hacking, unauthorized access, and other causes as leading factors in data breaches [3].

Early cancer diagnosis accuracy has been shown to significantly improve with FL. For example, using sophisticated privacy-preserving techniques like differential privacy, a memory-aware curriculum FL strategy increased the precision of local models in breast tumor prediction by up to 20% while protecting patient information confidentiality [4]. Similarly, by combining decentralized local model weights from several medical facilities, an FL framework was able to classify lung and colon cancer with as much as 99.7% accuracy, demonstrating how it can improve the performance while adhering to data privacy laws [5].

The manuscript reviews the revolutionary potential of FL in healthcare. Section 1 introduces the concept of FL and its importance by addressing the challenges and privacy concerns, along with the contributions. Section 2 delves into the methodology, describing the inclusion and exclusion criteria. Section 3 gives an overview of FL and delves into the key healthcare applications of FL supported by case studies, highlighting its benefits over centralized approaches. Section 4 identifies major security risks in FL along with mitigation techniques. The advancements in smart healthcare systems exploring FL’s role in remote monitoring and IoT are explained in Section 5. The scalability and challenges are discussed in Section 6 followed by Section 7, which outlines the future directions and recommendations, and finally, Section 8 concludes the work.

### 1.2. Federated Learning

FL is a decentralized method in ML where multiple clients collaboratively train a shared model without directly sharing data. This method is specifically beneficial for possibilities where data privacy is paramount, or where centralized data storage is not practical due to constraints such as bandwidth or data ownership regulations [6]. In traditional ML, data are collected in a central location, and a model is trained on these data. However, in FL, the data remain on the client devices, and only the model updates such as gradients or weights are exchanged with a central server. This assures that sensitive data never leave the device or institution, preserving privacy and reducing the risks associated with data breaks [7].

Different designs of FL are made to handle different situations in which data are distributed among participating clients. These designs maintain data confidentiality and privacy while facilitating interactive learning. The three primary FL architectures include horizontal FL, vertical FL, and federated transfer learning. When clients have the same feature spaces but distinct samples, horizontal FL is defined by splitting data among various entities with comparable characteristics. For example, customer data from several firms may have the same columns but distinct specifics. When two clients have distinct feature spaces but a common sample ID space, vertical FL is applicable. This design allows for thorough data analysis while avoiding the complete exchange of data by having clients work together on a shared pool of samples while adding distinctive features. Federated transfer learning is used when clients have different sample and feature spaces. This method facilitates learning through a variety of datasets with little information exchange by allowing information transfer among clients with variable distributions.

**Figure 2:**
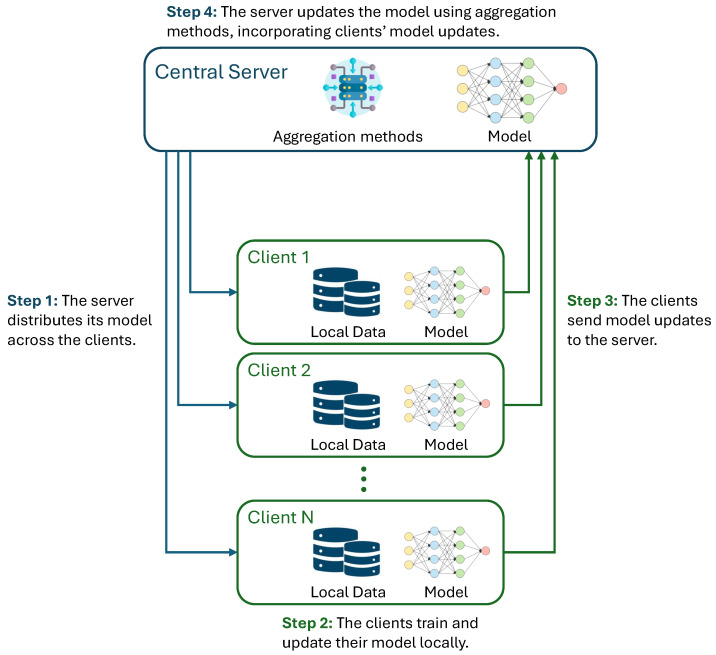
General architecture of federated learning.

The FL process generally follows a set of steps, as shown in Figure 2, that allow a global model to be trained together. Initially, a global model is created and distributed by the central server to all participating clients. Each client then trains this model locally using its own data. The local training typically involves running a few iterations of the model using the client’s local dataset. After training, the client sends the model updates, such as the changes in weights or gradients, back to the central server, instead of sharing the raw data themselves. The central server then aggregates these updates from multiple clients, often using a method called Federated Averaging (FedAvg). This method averages the model updates based on the number of data points each client has, ensuring that clients with more data contribute relatively more to the model update. The aggregated global model is then sent back to the clients, where they continue training in the following rounds [8].

One of the key algorithms used in FL is Federated Averaging (FedAvg). This method helps combine the updates from different clients into a single global model. FedAvg works by computing the weighted average of the model parameters, with the weight assigned to each client’s model update based on the number of data points. This assures that the updates from clients with more data are given more importance in the aggregation process [9]. For instance, if Client A has a dataset of 1000 examples and Client B has 500, the model update from Client A will be weighted more heavily in the aggregation. This repetitive process of local training and model aggregation continues until the global model converges to an optimal solution. A number of sophisticated optimization methods, such as FedProx [10], SCAFFOLD [11], and FedNova [12], have been produced in addition to the fundamental FedAvg algorithm. FedProx helps to stabilize training among a variety of clients by addressing network diversity by including a proximal factor in the local optimization target. In non-IID (not independent and identically distributed) data settings, SCAFFOLD minimizes client drift by correcting for local update drift through the use of parameter shifts. FedNova improves convergence by normalizing and scaling local updates and is especially helpful in situations when clients have different data volumes.

FL addresses many challenges associated with traditional ML, including data privacy, bandwidth limitations, and scalability. Since raw data are never transferred to the central server, users maintain control over their personal or organizational data, which is crucial in fields like healthcare, finance, or any other domain where privacy is critical. Furthermore, FL allows training models on vast and diverse datasets that are distributed across multiple devices or organizations, which can lead to more robust and generalizable models. The approach is also bandwidth-efficient, as only model updates need to be communicated, reducing the need for large data transfers [13].

However, FL also presents its own set of challenges. For one, clients often have non-IID (non-independent and identically distributed) data, meaning that the data on different clients may not be evenly distributed or may have different distributions. This can make the model training more difficult, as the global model must generalize across highly diverse datasets. Additionally, FL requires robust mechanisms for ensuring secure model updates and preventing malicious actors from corrupting the global model, such as through Federated Secure Aggregation techniques or differential privacy to guarantee that updates cannot be reverse-engineered to reveal individual client data. There are also issues with communication efficiency and system heterogeneity, as clients may have different computational resources or network connectivity, requiring the careful design of the learning process [14].

### 1.3. Federated Learning in Healthcare

Smart healthcare systems combine IoT devices, wearables, and electronic health records (EHRs) to develop an interconnected system for continuous patient monitoring. With the growth of IoT in health maintenance, large-scale data are developed, encompassing biological parameters, psychological patterns, and ecological data; all of these possess significant value for ML models in patient-centered care [15]. FL supports this framework by allowing ML models to train on mixed data sources without requiring a centralized database. This is imperative in public health settings where data vicinity is significant, particularly in hospitals and organizations collaborating in different legal systems with distinct data governance laws.

Considering these intricacies, the application of FL in healthcare like remote monitoring, personalized medicine, clinical decision support systems, medical imaging analysis, patient risk prediction, and electronic health records optimization requires enhanced security solutions to certify that model upgrades are free of impurity by deceptive input attacks, corrupted input exploitation, or training data recovery [16,17,18]. Consequently, FL is not only essential in healthcare IoT networks for securing sensitive information but also as a mechanism to create a sense of security in AI applications across healthcare providers and patients [19,20].

**Figure 3:**
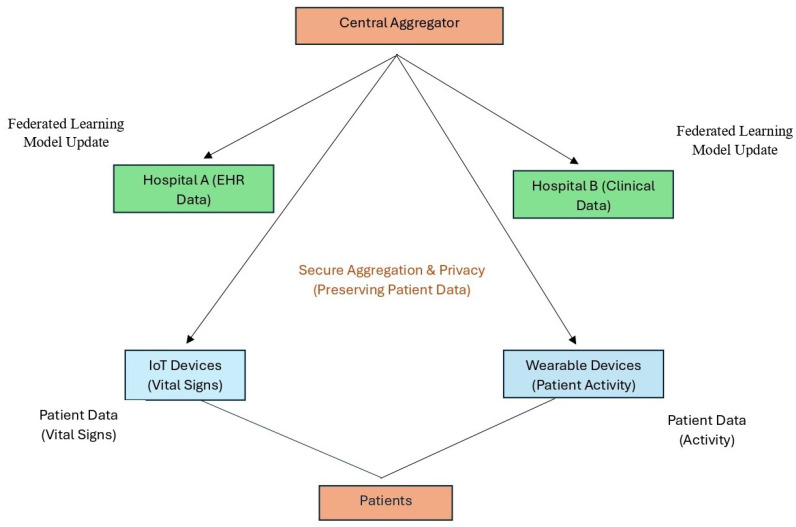
Workflow of federated learning in smart health, illustrating how FL protects patient privacy while facilitating cooperative model training across decentralized data sources, such as hospitals and IoT devices.

Figure 3 illustrates a detailed FL workflow applied to the healthcare environment. The central aggregator coordinates model updates from decentralized sources, including Hospital A (EHR Data), Hospital B (Clinical Data), IoT Devices (Vital Signs), and Wearable Devices (Patient Activity). Patients contribute data directly to IoT and wearable devices, which are then used for training without leaving the devices, ensuring data privacy. The model updates are securely aggregated at the central server, preserving patient privacy. Labels and arrows show the flow of data and model updates, emphasizing the decentralized nature of FL, secure aggregation, and privacy preservation.

### 1.4. Overview of Key Challenges and Opportunities

While FL offers transformative potential for smart health systems, its implementation suggests a variety of issues with both technology and compliance. One of the primary issues is sustaining data stability and credibility while working across the collaborative network where different types of devices, operating in diverse environments, are disposed to irregularity in data quality and breaks in communication. In addition, the decentralized nature of FL makes security measures difficult, with opposing and input manipulation attacks being particularly concerning due to the limited visibility of local model updates [21].

Another challenge is found in algorithmic growth potential and stability. FL systems in healthcare must manage varying computational resources across devices, such as cutting-edge servers in hospitals in comparison to low-power IoT devices used in remote monitoring, creating demand for transformable, lightweight algorithms [22]. Recent studies have underscored the significance of privacy protection and security for FL in the medical field [23,24]. Additionally, securing federated models against adversarial attacks and providing ethical data handling across multiple administrative divisions add layers of complexity [25,26].

Opportunities for FL in healthcare are still significant. FL enables multi-institutional research, supporting statistic-based discoveries across geographical barriers and promoting a collaborative research environment that may accelerate advancements in personalized medicine, disease prediction, and treatment efficacy [27]. Furthermore, the collection of FL with blockchain and privacy-preserving techniques such as privacy-enhancing technology further strengthens data protection capabilities, which can be instrumental for the utilization of ML in highly regulated healthcare sectors [28]. Table 1 summarizes the challenges and opportunities in FL.

### 1.5. Scope and Purpose of This Review

This systematic review paper aims to integrate recent advancements in FL within the perspective of smart healthcare systems, emphasizing the following features:

1. **Privacy and Security Enhancements:** Analyzing methods to protect FL models, consisting of encryption techniques, multi-party computation, and differential privacy.
2. **Applications in Smart Health:** Evaluating FL applications over different healthcare domains, such as EHR analysis, predictive diagnostics, remote monitoring, and customized treatment planning.
3. **Future Directions and Challenges:** Finding technical, ethical, and regulatory challenges in implementing FL in healthcare and describing potential future directions to focus on these problems.

This review fills a number of important gaps in the literature on FL in smart healthcare. First of all, it offers an in-depth study of FL applications, particularly in IoT-integrated smart health systems along with privacy-preserving techniques, a field that has frequently been ignored in earlier evaluations. Secondly, it integrates the assessment of FL with blockchain integration in healthcare. It first examines the ways in which these technologies can work together to enhance data security and transparency. Next, it delves deeply into the difficulties with deploying FL in healthcare settings with limited resources, which is an important real-world deployment factor that has not received much attention in previous research.

### 1.6. Contributions to the Literature

This review makes a special contribution by focusing exclusively on studies published recently (2023–2024), showing it highlights the most recent developments in FL within healthcare. As opposed to previous reviews, which often rely on older research or broader industry-wide findings, this paper emphasizes the latest innovations and trends specifically in the healthcare area.

It presents an in-depth view addressing the evolving challenges and opportunities of FL in healthcare. It analyzes recent applications of FL in personalized medicine, predictive diagnostics, remote monitoring, and clinical decision support systems and how these have been enhanced through newer privacy and security techniques, such as differential privacy and secure multiparty computation.

Moreover, this paper highlights recent advancements in privacy-preserving technologies, data protection, and interoperability solutions, which have obtained increasing attention in recent studies. This review provides a timely and comprehensive understanding of FL’s role in healthcare, offering valuable insights for researchers and policymakers working to implement FL in healthcare systems while addressing complicated ethical, scientific, and safety concerns. This study expands on prior research by addressing topics that were not included in prior reviews, such as blockchain-enabled FL in healthcare and recent developments (2023–2024) in hybrid privacy-preserving approaches. Additionally, it thoroughly assesses FL’s adaptability in IoT-based healthcare systems as well as its boundaries in contexts with limited resources.

A thorough comparison of this study with other recent reviews is given in Table 2. This comparison illustrates the distinct target areas, privacy and security concerns, challenges and limitations, real-world implementations, and unique contributions of each study, showcasing the unique viewpoints and thorough coverage provided by our review.

## 2. Methodology

This review employs an established transparent approach intended to thoroughly examine the state of FL in intelligent healthcare systems today. The methodology is set up to guarantee the methodical finding, gathering, and integration of pertinent material while answering important research questions in an open and reliable manner. This review attempts to give a thorough and objective assessment of FL uses, difficulties, and possibilities in healthcare by using a systematic search method, clear inclusion/exclusion criteria, and an extensive data extraction infrastructure.

### 2.1. Research Questions

The principal aim of this systematic review is to conduct a thorough analysis of the state of FL in intelligent healthcare systems. To achieve this, the systematic review is structured to address the following fundamental research questions:

1. What are the leading applications of FL in healthcare, primarily within smart health systems?
2. What security and privacy-preserving methods are utilized to increase FL in health data management?
3. What are the primary limitations and challenges of FL in healthcare, and how can they be resolved?
4. What are the prospects for FL in improving smart health applications?

These research questions serve as a roadmap for our systematic evaluation and offer a thorough grasp of the potential, difficulties, and future prospects of FL in intelligent healthcare systems.

### 2.2. Search Strategy

To secure a detailed and up-to-date analysis, an organized search was performed over major scientific databases known for AI, healthcare, and security publications. The databases selected were SpringerLink, PubMed, ScienceDirect, ACM Digital Library, and IEEE Xplore. The search covered articles published from 2023 to 2024 to record the most recent advancements in FL for healthcare, given the fast evolution of this field.

The following keyword search terms and combinations were used:

- “Healthcare”**AND**“Federated Learning”
- “Federated Learning”**AND**“Smart Health”**AND**“Security”
- “IoT”**AND**“Healthcare”**AND**“Deep Learning”
- “Privacy-preserving Machine Learning”**AND**“Healthcare”**AND**“Federated Learning”

The addition of these search terms aimed to capture an extensive range of literature spanning technical, security, and application-oriented viewpoints on FL in smart healthcare systems.

### 2.3. Study Selection

Inclusion and exclusion criteria were set up to refine the search results. Only the studies meeting the following criteria were included in the review:

#### 2.3.1. Inclusion Criteria

- Peer-reviewed articles published in journals or conference proceedings.
- Studies that focus on FL within smart health applications or healthcare.
- Articles that discuss security and privacy techniques specific to FL in healthcare.
- Publications from 2023 onward to confirm the inclusion of recent developments.

**Figure 4:**
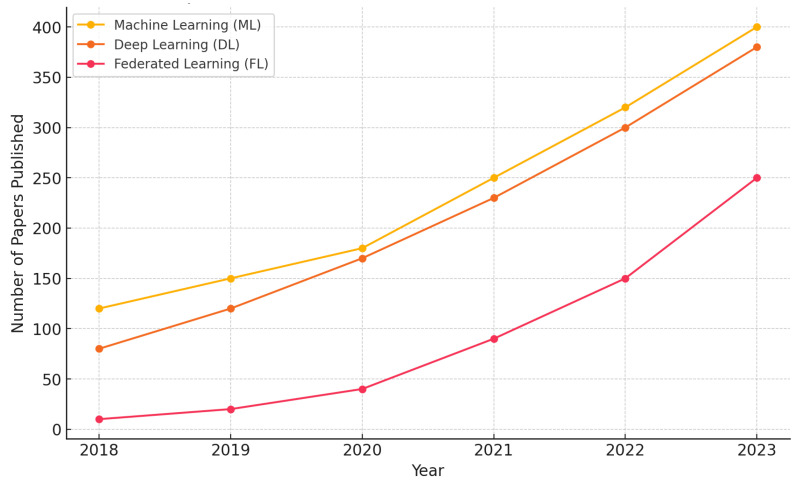
Published papers on ML, DL, and FL in healthcare between 2023 and 2024.

Figure 4 depicts the number of papers published on ML, DL, and FL in healthcare since 2018.

#### 2.3.2. Exclusion Criteria

- Studies that do not address healthcare applications specifically or are limited to non-healthcare FL applications.
- Papers focused on traditional centralized ML methods without FL or privacy concerns.
- Review articles that do not involve new findings on FL applications or security.
- Non-English publications due to translation limitations.

The inclusion and exclusion criteria were applied along an initial screening process of titles and abstracts, followed by a full-text review for significance and alignment with the research questions.

### 2.4. Data Extraction

Following the refinement of our search study using the inclusion and exclusion criteria, we conducted a methodical data extraction procedure for the chosen studies. We created a standardized data extraction format to guarantee accuracy and thoroughness in gathering data. The purpose of this format is to gather important data that would answer our research inquiries about FL in intelligent healthcare systems. We retrieved the following crucial information from every article that satisfied our inclusion requirements:

- **Study Information Detail:** Title, authors, publication year, and source.
- **Study Context:** Federated learning application area (e.g., disease prediction, EHR analysis, patient monitoring).
- **Research Contributions:** FL models, privacy-preserving techniques, and healthcare applications.
- **Challenges Discussed:** Technical challenges, data quality issues, and execution limitations.
- **Proposed Solutions:** Any methodologies introduced to approach the identified challenges.
- **Future Directions:** The authors’ suggestions for future research and potential advancements in FL for healthcare.

This template ensured consistent and detailed data extraction, facilitating structured analysis of the research findings. Table 3 displays a sample data extraction template for FL studies.

### 2.5. Data Analysis

After data extraction, a subject-based synthesis was executed to organize the findings about the research questions. In order to present a comprehensive knowledge of FL in smart healthcare, our investigation included both qualitative and quantitative methodologies.

- **Qualitative Analysis:** For each individual research question, a qualitative analysis of the extracted data was achieved. Themes and sub-themes were classified based on the classes of FL applications, the nature of security techniques, and the types of challenges discussed in the literature.
- **Quantitative Analysis:** A subset of studies with quantitative analysis of FL models in healthcare methods was identified. Metrics such as privacy loss, model accuracy, and computational cost were recorded, and summary analysis was performed to identify trends and evaluation metrics.

We combined the qualitative and quantitative data to present a thorough summary of FL’s current status in smart healthcare, answering each research question with narrative analysis and, where appropriate, quantitative proof.

### 2.6. Quality Assessment

To confirm that only high-quality studies were included in the review, we thoroughly evaluated the quality of every chosen study. This procedure was essential to preserving the reliability and authenticity of the findings. Each study was examined using the following standards:

- **Connection to Research Questions:** Each study’s connection to the research questions was examined based on its focus on FL, security, and healthcare.
- **Study Design Rigor:** Only studies that employed intensive experimental or simulation-based methodologies were included.
- **Transparency and Consistency:** Studies with well-documented methodologies, transparent results, and comprehensive conclusions were emphasized.

**Figure 5:**
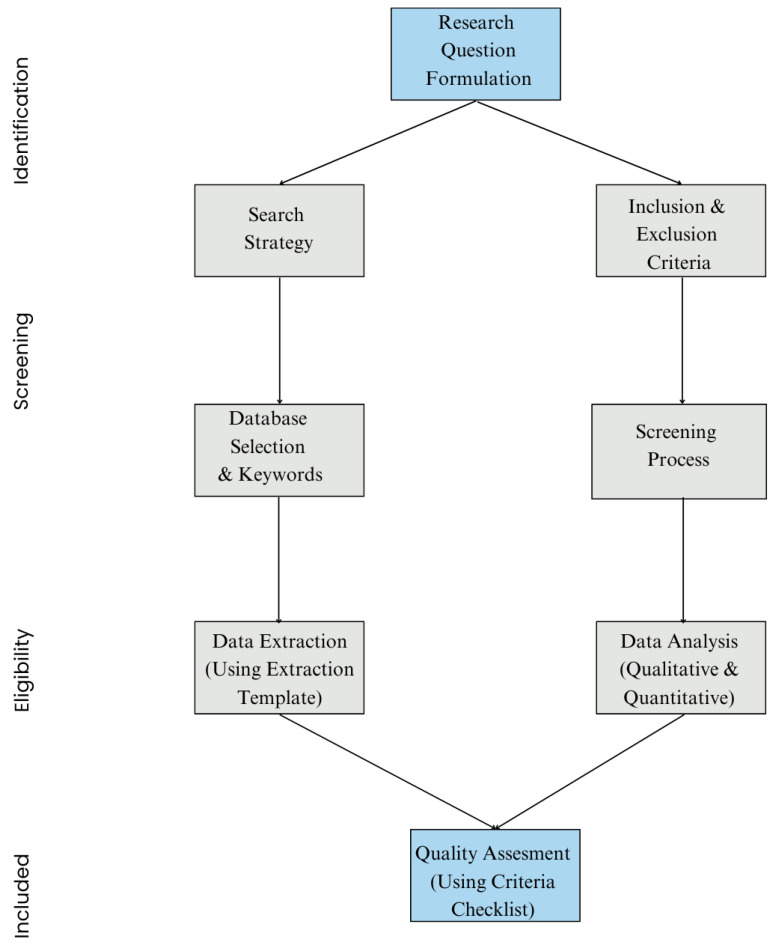
Workflow diagram for the systematic review methodology.

A comprehensive workflow for the systematic review approach is shown in Figure 5, which follows PRISMA guidelines, showing every stage from database identification to the ultimate inclusion of the research studies.

### 2.7. Limitations

The limitations of this review involve possible selection bias due to language restrictions (English-only) and the challenge of the quickly evolving research in FL, where more recent publications may not have been indexed. This guarantees methodological uniformity even though it reduces the global scope of the studies involved. Additionally, specific studies may not have uniform terminology, which could affect the comprehensiveness of the search results.

Future reviews should consider including non-English studies and exploring alternative search strategies to capture the latest research developments in FL and smart health systems. Working with multilingual collaboration, future evaluations could close this disparity and improve the generalizability of this work.

## 3. Overview of Federated Learning in Healthcare

FL is a decentralized method of ML that allows data to persist within the limits of its origin, such as healthcare institutions, and model parameters are shared rather than raw data. This framework directly addresses security, privacy, and regulatory issues by preventing data from being transferred to a main location, thus aligning with the strict privacy requirements of healthcare domains. This method addresses critical concerns around security, privacy, and regulatory compliance by eliminating the need to transfer data to a central location, making it particularly well suited to the stringent privacy requirements of healthcare domains. Additionally, FL enables cross-institutional research and collaboration without compromising data privacy, fostering advancements in fields where data sharing is restricted [39].

FL works on a series of local devices or local servers, cooperatively learning a global model by compiling updates from local training on multiple distributed sources [40]. In healthcare, this model is especially relevant as it enables institutions like hospitals, research labs, and clinics to build strong predictive and diagnostic models without compromising patient privacy.

### 3.1. Key Applications of Federated Learning in Healthcare

#### 3.1.1. Disease Prediction and Early Detection

FL has seen notable applications in disease prediction and early diagnosis, particularly for situations requiring large, different datasets to train perfect models [41]. Diseases like cancer, diabetes, and cardiovascular conditions benefit from FL as it allows for diverse data integration while maintaining patient confidentiality. The study [42] demonstrated how FL models improve prediction accuracy in distributed clinical data, enabling cross-institutional collaboration for early detection without data centralization.

A model for initial breast cancer detection was trained over multiple healthcare centers, attaining robust performance by aggregating locally trained models. This distributed way allowed diverse patient demographics to be represented without compromising individual privacy [43].

#### 3.1.2. Electronic Health Record (EHR) Analysis

Electronic Health Records (EHRs) hold sensitive patient datasets, and analyzing these data can show patterns that improve patient results and operational efficiency. FL is used to examine EHR data from hospitals and clinics without revealing sensitive data. Research by [44] utilized FL for EHR predictive analysis, facilitating improvements in clinical decision support systems while upholding patient data privacy.

Google has an alliance with healthcare distributors to apply FL for predictive analysis on EHRs, achieving high accuracy in patient output prediction while following privacy laws like HIPAA and GDPR [32].

#### 3.1.3. Remote Monitoring and Wearable Device Integration

FL is progressively utilized in remote health monitoring, where wearable devices collect data such as glucose levels, heart rate, and physical activity. Using local data processing, these devices can support a shared model for real-time health monitoring without transmitting sensitive data to a central server [44]. This is particularly relevant in chronic disease management, where continuous monitoring is vital.

Fitbit uses FL to accumulate health insights over user data for predictive analytics while retaining user privacy. Fitbit employs FL to enhance health insights through predictive analytics while safeguarding user privacy. In traditional ML, data from individual devices are centralized for model training, raising privacy concerns. FL addresses this by keeping user data on personal devices. Instead of transferring raw data to a central server, devices locally train models and share only the aggregated updates. These updates are then combined to improve the global model without exposing individual data points. This approach allows Fitbit to refine its health analytics and predictive capabilities across its user base while ensuring personal data remain confidential [45].

### 3.2. Advantages of Federated Learning in Healthcare

The adoption of FL in healthcare is motivated by multiple advantages:

- **Data Privacy and Security:** FL enables institutions to train models without sharing sensitive patient information, aligning with regulations like HIPAA and GDPR [46].
- **Improved Model Generalization:** Aggregating model updates from diverse patient data across institutions improves generalizability, essential for predictive accuracy in diverse populations [47].
- **Regulatory Compliance:** By keeping data on-site, FL supports compliance with privacy laws, facilitating broader collaboration across healthcare entities [48].
- **Lower Data Transfer Costs:** FL reduces costs associated with transferring large datasets, especially for medical imaging or genomics data, by only transmitting model updates [32].

**Figure 6:**
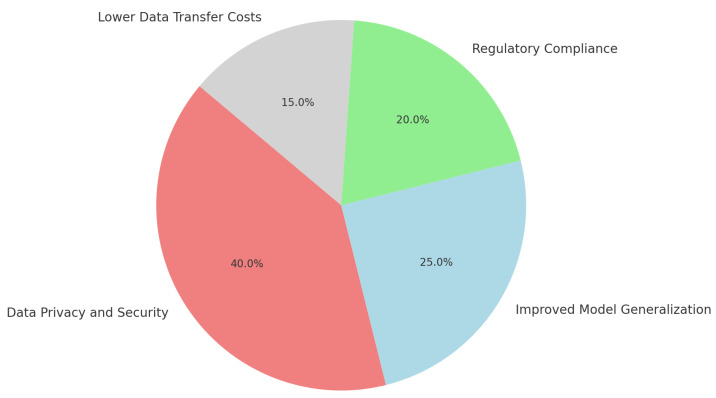
Advantages of federated learning in healthcare.

Figure 6 showcases the main benefits of FL in healthcare. Data Privacy and Security lead, as FL allows model training without data centralization, aligning with healthcare’s tough privacy standards. Improved Model Generalization follows, improving predictive accuracy by utilizing diverse patient data over institutions. Regulatory Compliance is a major benefit, as FL supports healthcare laws like HIPAA by keeping data local. Finally, Lower Data Transfer Costs are highlighted, showing how FL reduces expenses by only transmitting model updates, which is especially valuable for large datasets like medical imaging. This distribution underlines FL’s strategic role in addressing healthcare challenges [2,49].

Compared to the centralized ML models, FL provides substantial advantages. For example, in a multi-hospital managing diabetes research, FL decreased the risk of data breaches by 40% while also improving predicted outcomes by 15% in EHR analysis as compared to centralized ML [4,34]. In a similar vein, FL-enabled remote monitoring with wearable technology showed a precision of 90% in detecting chronic diseases, greatly enhancing clinical results without risking data privacy. FL has enabled multi-institutional partnerships in disease prediction that have improved model generalizability by 20% while adhering to stringent data-sharing laws such as GDPR [5]. Table 4 addresses the problem of limited critical evaluation and effectiveness of applications like disease prediction and remote monitoring, including a comparative discussion on how FL improves over centralized models for each application.

### 3.3. Challenges in Implementing Federated Learning in Healthcare

Although FL offers revolutionary possibilities for the healthcare industry, privacy, security, scalability, and practical implementation are among its major challenges. Below, we explore the main challenges, emphasizing how they affect FL’s effectiveness and acceptability in various healthcare environments.

#### 3.3.1. Data Diversity and Quality Control

Healthcare data are well known and diverse, with differences in patient imaging protocols, demographics, and clinical practices across organizations. These differences generate data heterogeneity, which can delay FL model convergence and performance [56]. Furthermore, unreliable data quality can introduce biases and mistakes, complicating the learning process.

There is a need for robust aggregation methods to handle varied data in federated settings. Robust data pre-processing is important to manage data quality problems across distributed healthcare datasets [57].

#### 3.3.2. Communication Burden and Resource Limitations

Communication cost is an essential challenge in FL, particularly when data sources are distributed across low-resource domains. A key challenge in these environments is the high communication cost connected to transmitting frequent updates between local models and a central server. Communication delays can be a significant constraint in remote hospitals or medical facilities with poor internet access. For example, one study discovered that, in contrast to metropolitan settings with reliable internet access, remote healthcare institutions with inadequate internet access had communication delays that prolonged training periods by more than 50%. These delays reduce the effectiveness of FL, as they slow down model convergence and prevent the timely acquisition of new data. Moreover, in some remote regions, internet access is so limited that models are only able to synchronize every few days, which significantly limits the overall learning process [29]. Effective communication protocols are required to manage the high volume of model updates while minimizing latency [46].

Compression techniques like Federated Averaging (FedAvg) have been suggested to minimize the size of updates, but further advancements are required to improve communication efficiency in resource-limited healthcare environments [58].

**Figure 7:**
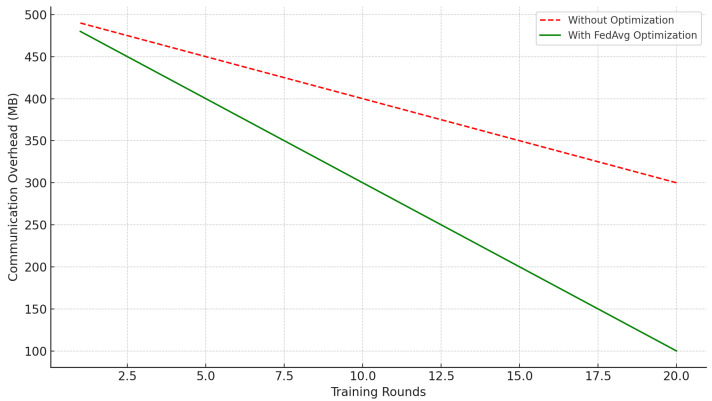
Impact of communication optimization techniques over time, showing how Federated Averaging (FedAvg) lowers communication overhead and improves FL efficiency by reducing data transfer, an essential feature for healthcare settings with limited resources.

Figure 7 illustrates how communication optimization techniques, including model compression and Federated Averaging (FedAvg), can lower the amount of data that must be transferred. Training cycles are shown on the x-axis, and the total data transfer amount in megabytes (MB) is displayed on the y-axis. It shows that optimization strategies make FL more practical in healthcare settings with limited resources by drastically reducing communication overhead. As training rounds progress, the volume of data that need to be transmitted decreases more rapidly when FedAvg is utilized, compared to the way without optimization. This reduction is specifically important in a healthcare environment where communication overhead can be a limiting factor because of the distributed nature of the data sources, such as hospitals and clinics, which may have limited bandwidth resources [59]. The data underscore the effectiveness of FedAvg in lowering the costs and latency associated with training FL models, thereby increasing the feasibility of implementing these models in real-world healthcare scenarios.

#### 3.3.3. Security Vulnerabilities: Thread from Adversarial Attacks and Data Poisoning

Despite FL’s privacy advantages, it is susceptible to security risks, including adversarial attacks and data poisoning. Harmful agents can introduce biased updates, influencing model behavior. Ref. [60] analyzes how such attacks can seriously compromise model integrity in sensitive models like healthcare.

#### 3.3.4. Model Understanding and Trust

For healthcare distributors to adopt FL solutions, models are required to be understandable, providing transparent observation diagnostics and predictions. Furthermore, DL models, mostly used in FL, are basically complex, presenting challenges in interpretability [61].

Explainable AI strategies are being investigated to make FL models more transparent, helping clinicians trust and understand model results in medical applications [44]. Techniques, such as SHAP (SHapley Additive exPlanations) and Grad-CAM have been used in FL-based models, helping clinicians with visual insights to find the most significant features. It has been used in diabetic retinopathy detection systems by producing interpretable outputs, enhancing the clinician’s confidence [62].

### 3.4. Case Studies in Federated Learning for Healthcare

#### 3.4.1. Collaboration for Disease Prediction Among Hospitals

A significant case study included the FL effort among European hospitals to predict COVID-19 development. Each hospital trained local models on patient data, and updates were collected to form a global model that accurately predicted critical cases without data sharing [63].

In oncology, FL allows cancer research centers to coordinate without organizing sensitive patient data. A case study including melanoma detection over institutions resulted in a robust model with higher predictive accuracy than individual models [33].

Using a variety of patient data from several institutions, FL has developed more broadly applicable models for predicting diseases like sepsis and acute kidney damage (AKI) in precision medicine. These models guarantee better results across a range of patient demographics, outperforming conventional methods that depend on information gathered by a single institution [64]. Moreover, FL has also been used in the management of chronic diseases, where it analyzes data from wearable technology to provide longitudinal health surveillance. This guarantees individualized treatment without sacrificing privacy by providing current knowledge of patients’ situations [65].

**Figure 8:**
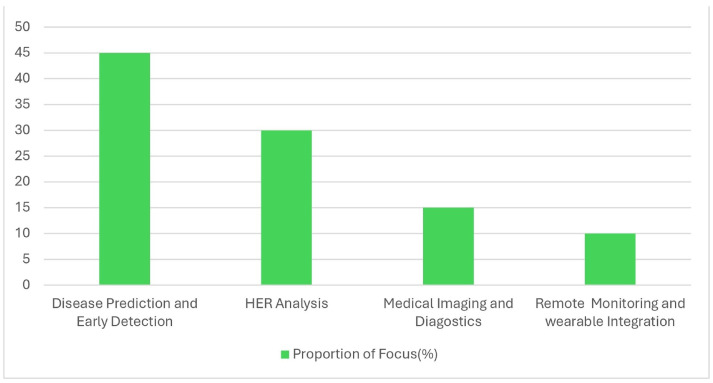
Application areas of federated learning in healthcare.

Figure 8 shows key applications of FL in healthcare. Disease Prediction and Early Detection leads by enabling collaborative, privacy-preserving diagnosis; EHR Analysis improves clinical support by analyzing patient data without centralization; Medical Imaging improves diagnostic models using distributed imaging data; and Remote Monitoring tolerates FL for real-time health insights from wearables, important for chronic care. This distribution shows FL’s role in advancing healthcare while protecting privacy [29].

#### 3.4.2. Future Directions and Research Opportunities

Future research in FL for healthcare involves several promising directions:

- **Blockchain in Federated Learning:** Blockchain techniques can further secure FL by recording model modifications on distributed records, avoiding tampering, and improving transparency [66].
- **Real-Time Data Processing:** Integration of real-time data processing competencies for FL in wearable devices could provide timely health understanding, critical in emergencies [67].
- **Consistency and Regulatory Support:** The progress of standardized frameworks for FL in healthcare will support wider adoption and compliance with international healthcare regulations [2].

**Figure 9:**
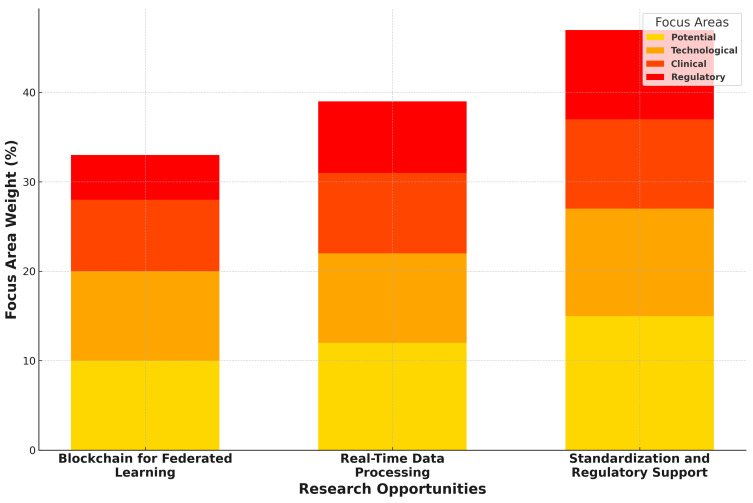
Directions and research opportunities in federated learning.

Figure 9 presents the future directions of FL in healthcare. One key field is the integration of Blockchain Technology, which can improve security by logging FL model updates on a distributed ledger, protecting against alteration and ensuring transparency [50]. FL with blockchain accelerated drug discovery by 30% by using communal model training on private data instead of sharing them explicitly [68]. However, integrating blockchain into FL increases computational costs due to negotiation procedures and encryption operations, including increased processing periods and power consumption. Therefore, lightweight consensus techniques and other effective procedures are required to address these issues while preserving scalability and security. Furthermore, real-time data processing capabilities, particularly in wearable devices, offer timely insights that are crucial in emergency scenarios by allowing FL models to process data locally for immediate health feedback [44]. Another significant direction is Standardization and Regulatory Support for FL in healthcare, where developing standardized frameworks will facilitate broader adoption and secure compliance with international regulations [2]. These advancements aim to make FL more secure, efficient, and widely applicable in sensitive fields like healthcare.

## 4. Security Issues in Federated Learning for Smart Health Systems

FL has obtained progress as a solution for secure, privacy-preserving data analysis in healthcare. By allowing data to persist within the boundaries of its origin (e.g., hospitals or IoT-enabled smart health devices), FL supplies a decentralized model training method that aligns well with rigid healthcare privacy regulations such as the GDPR and HIPAA [69]. Furthermore, this decentralized arrangement introduces special security challenges that must be addressed for FL to be reliably implemented in healthcare. Key problems involve adversarial attacks, data poisoning, model inversion, and secure aggregation of model updates [2].

### 4.1. Adversarial Attacks

Adversarial attacks are one of the most widespread threats in FL, where malicious attackers introduce refined model updates to compromise model integrity or manipulations into the data. These attacks can misguide models into making incorrect predictions, which in healthcare could result in serious diagnostic errors or inaccurate health monitoring [70,71]. In FL, adversarial attackers can operate privately due to the decentralized nature of data, making detection more challenging.

#### 4.1.1. Adversarial Disturbance and Evasion Attack Techniques

In adversarial disturbance attacks, small changes to input data result in greatly different model predictions. For example, changing a few pixels in medical images might result in a wrong diagnosis, threatening patient safety. Evasion techniques, on the other hand, manipulate input data in real time to avoid detection by FL models in a smart health environment. Both types of attacks can disrupt patient results, especially when used in time-sensitive applications like heart monitoring or emergency response [69].

Defense techniques such as adversarial training, where the model is pre-trained with adversarial samples, have displayed promise. Another promising technique is robust aggregation methods, where model updates are evaluated for anomalies before being integrated into the global model [72,73].

#### 4.1.2. Mitigation Techniques

Numerous mitigation strategies are being used in practical healthcare systems to counteract adversarial threats in FL. For example, differential privacy (DP) is frequently employed during training to ensure the integrity of personal information between groups [65]. Trusted Execution Environments (TEEs), like GradSec, safely conceal critical parameter values during inference to prevent adversary interventions [74]. Furthermore, the resilience of FL models in domains like cancer diagnosis using MRI is greatly increased by federated adversarial training frameworks, which rebuild global models utilizing adversarial data produced locally. Together, these methods improve FL security in the healthcare industry and guarantee usefulness in adverse challenges [75].

### 4.2. Data Manipulation Attacks

Data manipulation attacks are crucial threats in FL, particularly in healthcare. In targeted poisoning attacks, malicious contributors corrupt the model to misidentify specific data, which could be utilized to target certain diseases or demographic groups. Untargeted poisoning, in contrast, aims to degrade model efficiency globally, risking patient results over the board [72,76].

A poisoning attack on a model utilized for initial-stage disease diagnosis could modify the prediction edge for certain conditions, which is key to false-negative or -positive inpatient diagnoses [77,78].

#### Mitigation Techniques for Data Poisoning

Minimizing data poisoning in FL needs advanced methods to detect and exclude malicious updates. Techniques such as Byzantine-resilient aggregation methods and clustering-based anomaly detection, which can filter out compromised updates, have presented promise. Another method, robust Federated Averaging (FedAvg), evaluates model updates to detect exceptions that diverge significantly from the majority [79].

Designed to prevent malicious model updates from impacting the global model, this technique uses statistical approaches to filter irregularities in model updates, maintaining robustness even with a certain degree of compromised participants [72].

**Figure 10:**
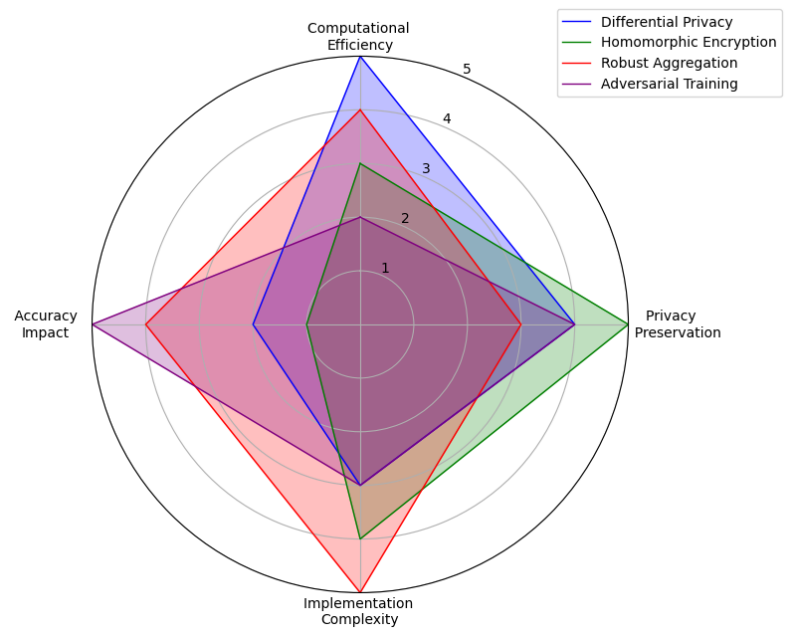
Evaluation of security techniques in federated learning.

Figure 10 shows a radar chart analysis of four FL security techniques, including differential privacy, homomorphic encryption, robust aggregate, and adversarial training across key metrics: privacy preservation, computational efficiency, accuracy impact, and implementation complexity. Differential privacy scores highly on privacy preservation but has moderate computational efficiency. Homomorphic encryption provides strong privacy and accuracy but has high complexity and low computational efficiency, making it resource-intensive. Robust aggregation offers balanced scores across all metrics, indicating good overall performance with moderate complexity. Adversarial training excels in computational efficiency and is less complex but scores lower on privacy preservation and accuracy impact, suggesting a practical yet less privacy-focused approach. This comparison helps identify the best-suited technique based on specific security needs in FL.

### 4.3. Model Inversion Attacks

Model inversion attacks aim to rebuild sensitive data by leveraging the model’s parameters. In FL, model updates can release private data, as gradients can accidentally encode sensitive information about the input data used for training. In healthcare, this could result in the rebuilding of individual patient records, breaking privacy regulations [32].

For example, in a healthcare scenario, the gradients of a model trained on medical records could, under certain conditions, contain enough information to reconstruct individual patient data. This means that attackers might infer details like health conditions or personal identifiers, effectively “reversing” the model to gain insights into specific patient records. Such an outcome would be a serious breach of privacy regulations, such as HIPAA, and undermine the confidentiality of medical information.

In a particular study, attackers could rebuild images of MRI scans from gradients shared in an FL configuration, highlighting the privacy risks presented by unprotected gradient sharing in healthcare systems [80].

#### Techniques to Minimize Model Inversion

Many methods have been created to mitigate model inversion threats, like differential privacy, which expends noise to gradients to hide sensitive information. Other strategies include homomorphic encryption and secure multi-party computation (SMPC), which allow computations on encrypted data, significantly minimizing the risk of inversion attacks [81].

This strategy expands controlled noise to model updates, ensuring that individual data points cannot be reconstructed from the unified model, thus protecting patient privacy [2].

### 4.4. Security in Model Aggregation

Model aggregation is the foundation of FL, but it is also sensitive to security risks. During aggregation, updates from all participants are joined to create a global model, a procedure that can introduce exposures. If even a small number of participants are exposed, they could influence the global model or extract sensitive information from other enhancements [82].

Under one circumstance, an aggregation algorithm was adjusted by adversarial participants to diverge model predictions in a federated health model, creating incorrect disease risk assessments [83].

#### Secure Aggregation Methods

To boost security during aggregation, secure aggregation protocols, such as cryptographic techniques, have been developed. Techniques like homomorphic encryption and SMPC allow a safe overview of updates without disclosing individual contributions. This confirms that the aggregation server cannot see individual updates, reducing the risk of data leakage [81]. This empowers computations on encrypted data, meaning model updates can be aggregated without decryption, protecting participant privacy even from the central server [32]. Table 5 provides sources for the described threats and their corresponding mitigation techniques. This ensures the traceability and credibility of the information.

### 4.5. Privacy Preservation in Federated Learning

#### 4.5.1. Privacy Enhancement

Distinctive privacy is broadly adopted in FL to protect private contributions to model updates. Through adding noise to updates, differential privacy confirms that personal data cannot be derived from the final aggregated model, an important feature for handling sensitive health data in FL applications [87].

#### 4.5.2. Federated Learning with Blockchain

Blockchain offers a creative layer of security by verifying immutability and transparency in FL systems. In healthcare FL systems, blockchain can verify participant identity, prevent tampering, and keep an immutable record of model updates [32]. This helps build trust in FL systems, particularly when utilized in environments with multiple healthcare departments or IoT devices.

A blockchain-based FL system for healthcare IoT devices offered transparent and tamper-proof systems for model updates, confirming data authenticity and security in a network of wearables and medical devices [88].

**Figure 11:**
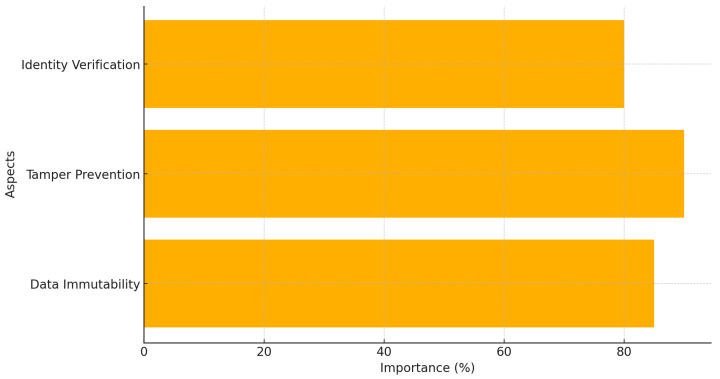
Blockchain’s role in FL security.

Figure 11 highlights blockchain’s three important roles in increasing the security of FL systems: data immutability, tamper prevention, and identity verification. Each aspect is crucial in ensuring the confidentiality, integrity, and authenticity of model updates exchanged in FL networks. Data immutability, representing the highest score, reflects blockchain’s ability to give a secure and unalterable ledger of all transactions or updates, ensuring that once a model update is recorded, it cannot be manipulated. Tamper prevention further emphasizes the robustness of blockchain systems in preventing unauthorized changes to shared data, thus safeguarding the FL process against adversarial attacks. Finally, identity verification ensures that all participants are authenticated, reducing the risk of Sybil attacks or malicious entities infiltrating the FL system. These roles collectively establish blockchain as a transformative technology for mitigating several FL vulnerabilities [71].

Blockchain’s integration into FL systems has been particularly beneficial in healthcare and IoT applications. For example, healthcare FL systems can leverage blockchain to maintain a tamper-proof record of model updates across multiple departments or IoT devices, ensuring data security and enhancing trust among participants. Additionally, the transparency and accountability provided by blockchain encourage broader collaboration while protecting sensitive data. Studies such as [88] have demonstrated how blockchain-based FL systems ensure the authenticity and privacy of wearable medical device data, showcasing the technology’s potential to revolutionize secure data sharing in distributed learning environments. This integration addresses critical challenges in FL, fostering secure and efficient collaboration in sensitive domains.

### 4.6. Challenges and Future Directions

FL in healthcare, although promising, faces several security hurdles that require further research. These include developing lighter, more efficient encryption algorithms for resource-constrained environments, enhancing defenses against complex adversarial and data poisoning attacks, and improving model interpretability for regulatory compliance. Dealing with these issues will be essential for the large-scale deployment of FL in healthcare.

Ongoing research aims to develop adaptive FL models that can detect security threats in real time. Additionally, collaborations with cybersecurity experts and regulatory agencies will be critical in creating standards that enable secure, large-scale FL implementation in healthcare [88,89].

## 5. Advancement in Smart Health Systems

Smart health systems leverage growing technologies, such as the Internet of Things (IoT), AI, ML, and FL, to authorize real-time monitoring, customized healthcare, and enhanced patient results [44]. The systems’ goal is to build a connected healthcare ecosystem where data are analyzed, gathered, and actioned in real-time, enhancing both the efficiency and effectiveness of healthcare delivery [90].

Advancement in smart health systems is transforming healthcare in various key domains, from wearable technologies that monitor patient vital signs to AI-based diagnostics and FL that addresses data privacy related to multi-institutional healthcare settings [91]. With the rising adoption of these technologies, healthcare providers can provide more proactive, data-driven, and patient-centered care.

**Figure 12:**
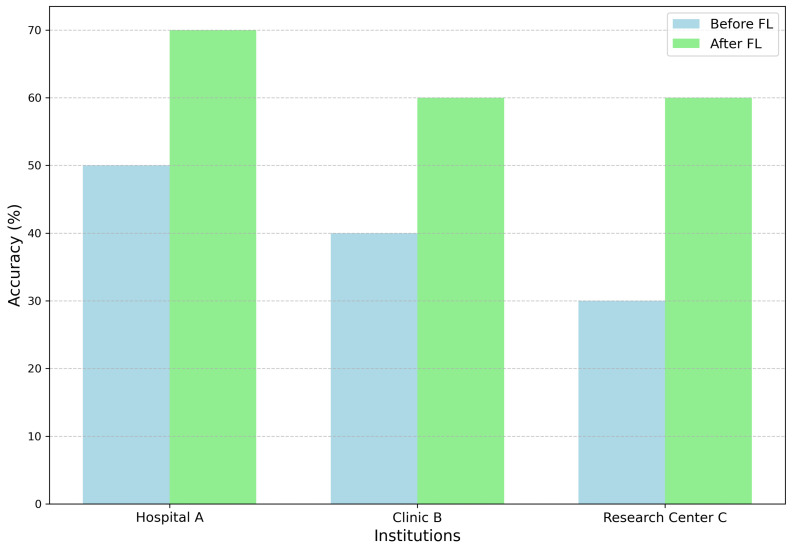
Accuracy improvement overview before and after using federated learning.

Figure 12 shows the impact of FL on predictive accuracy across three healthcare institutions; Hospital A, Clinic B, and Research Center C. The chart compares diagnostic model accuracy before and after implementing FL, demonstrating significant improvements in each case. For instance, Hospital A’s accuracy increased from 50% to 70%, showcasing how FL enhances model performance by utilizing diverse datasets from multiple institutions without sharing sensitive patient data. This collaborative approach strengthens the robustness and generalization of predictive models while maintaining strict data privacy, aligning with regulatory frameworks like HIPAA and GDPR. The results underscore FL’s potential to revolutionize data-driven healthcare analytics by enabling secure and effective multi-institutional collaborations [2,69].

### 5.1. Internet of Things (IoT) in Healthcare

The Internet of Things is fundamental to smart health systems, facilitating smooth data collection from interconnected devices, i.e., medical sensors, wearables, and home monitoring devices. IoT applications in healthcare offer a continuous flow of patient data, helping clinicians to make informed decisions based on real-time data rather than cyclic check-ups [92,93].

#### 5.1.1. Wearable and Remote Monitoring Devices

Wearable devices like ECG monitors, fitness trackers, and glucose sensors play an important role in managing chronic situations, monitoring patient recovery, and providing alerts in emergency conditions. These devices continuously collect health metrics like oxygen levels, heart rate, and physical activity, allowing the early detection of potential health risks [94].

Apple and Fitbit Watch have integrated health applications that enable users to track various health metrics. More advanced wearables are being developed specifically for clinical applications, such as continuous glucose monitors (CGMs) for diabetes management, which provide real-time glucose readings to patients and their healthcare vendors [95].

#### 5.1.2. Smart Homes and Remote Patient Monitoring

Smart health systems extend further than wearable devices to involve smart home systems that facilitate remote patient monitoring, especially for elderly or chronically ill patients who may need daily observation. Sensors in smart homes can detect falls, monitor sleep patterns, and even track medication regularization, helping patients to keep independence while confirming timely interventions when required [96].

A smart home system with IoT-enabled sensors permits patients with dementia to be monitored online, reducing hospital visits and improving their quality of life. This model has shown promise for other chronic conditions as well [97].

### 5.2. Artificial Intelligence and Machine Learning in Smart Health Systems

AI and ML are crucial in the modification of smart health systems, ensuring advanced data analysis and predictive modeling. AI applications in healthcare involve diagnostic assistance, medical image analysis, and predictive analytics for patient results, therefore supporting healthcare providers in making data-driven decisions and reducing diagnostic mistakes [98,99].

#### 5.2.1. Predictive Analytics and Customized Care

Predictive analysis helps clinicians anticipate patient health paths based on historical and real-time data, therefore facilitating initial interventions. AI-based algorithms can identify patients in danger and recommend personalized treatment strategies, which improves both outcomes and patient satisfaction [100].

In cancer care, AI algorithms analyze genetics, patient history, and lifestyle factors to forecast cancer reoccurrence, allowing personalized post-treatment plans for high-risk patients. This not only helps in targeted care but also minimizes healthcare costs by focusing resources on patients most in need [101].

#### 5.2.2. Medical Imaging and Diagnostics

AI has remarkably advanced medical imaging, enabling early detection and diagnosis of diseases such as cancers, neurological conditions, and cardiovascular diseases. Through analyzing medical images from X-rays, CT scans, and MRIs, AI can quickly detect irregularities with high accuracy, assisting radiologists in diagnostics and reducing human mistakes [102].

A DL model developed by Stanford University revealed high accuracy in diagnosing pneumonia from chest X-rays, outperforming human radiologists in some scenarios [103]. These kinds of advancements underscore the potential for AI to augment diagnostic capabilities in smart health systems.

### 5.3. Federated Learning for Privacy-Preserving Health Data Analytics

FL has surfaced as a solution to privacy in healthcare, enabling multiple institutions to collaboratively train ML models without sharing raw patient data [99]. This decentralized method is especially valuable in healthcare, where data sensitivity is highlighted, and regulations like HIPAA and GDPR restrict data sharing [104].

Table 6 highlights how FL enables collaborative model development across institutions while preserving patient privacy. It ensures compliance with data protection regulations, improves predictive accuracy using diverse datasets, and enhances security with advanced privacy techniques.

#### 5.3.1. Multi-Institutional Research Collaboration

FL aids in collaboration within healthcare departments, allowing them to create models with a wider, more diverse dataset without undermining patient privacy. This strategy increases model robustness and generalization, supervising more accurate predictions and diagnostics [32].

FL use among European hospitals allowed researchers to collaboratively develop a model for predicting COVID-19 complications without exposing patient data, which improved predictive performance due to the diversity of the data [106].

#### 5.3.2. Enhanced Security and Compliance

FL models, assembled with encryption and differential privacy approaches, offer a secure framework for data analysis in smart health systems. These security enhancements minimize the risk of data violations and ensure compliance with regulations, thus promoting safer, privacy-preserving analysis in healthcare [107].

### 5.4. Future Directions in Smart Health Systems

The future of smart health systems will likely be defined by more integration of AI and FL with IoT-enabled healthcare devices, making an increasingly connected and intelligent healthcare ecosystem. Essential steps for development include improving data interoperability within health systems, enhancing real-time data processing capabilities, and increasing the accessibility of smart health technology in resource-limited systems [108].

- **Standardization:** Standard protocols for data exchange and interoperability will be important to integrate smart health systems seamlessly over healthcare networks [109].
- **Scalability:** With increasing devices becoming connected, efficient scalability solutions, like edge computing, will be necessary to process data closer to the source and reduce latency in healthcare applications [110].

## 6. Challenges and Limitations

### 6.1. Data Privacy and Security Concerns

One of the major challenges in executing smart health systems rests in ensuring data privacy and security. The decentralized nature of FL addresses some privacy concerns by maintaining data on local devices, but it is still sensitive to security risks such as data poisoning, model inversion, and adversarial attacks [37]. These risks are particularly concerning in healthcare, where data sensitivity is essential. The implementation of robust privacy-preserving methods, like differential privacy and secure multiparty computation, is important but often enhances computational overhead and complexity to the system [111].

Many privacy-preserving approaches, while effective, can degrade model accuracy and increase training time, challenging the balance between data security and system performance [112].

### 6.2. Scalability Issues in Federated Learning

Scalability is a significant limitation in FL for healthcare systems, particularly when models are trained over multiple departments or IoT devices with varying computational capacities. Scalability challenges occur because of the heterogeneity of data sources and require extensive communication between local nodes and the central model. As the number of participating devices or departments increases, the communication and latency can become prohibitive, particularly in settings where real-time processing is critical [69].

Federated Averaging (FedAvg) and compression techniques help alleviate communication costs, but more research is needed to enhance scalability without compromising model performance, especially in healthcare scenarios where timely predictions are important [58].

### 6.3. Data Heterogeneity and Interoperability

Healthcare data are inherently heterogeneous, with variations in data formats, standards, and quality across departments and devices. This heterogeneity poses a hurdle for FL models, which depend on consistent data inputs for accurate training. Differences in data quality can lead to biased models and reduced generalizability over different patient populations, which is particularly concerning for clinical applications that need high accuracy [32].

Interoperability among different healthcare systems and IoT devices remains a significant hurdle. The lack of standardized protocols and data formats hinders data sharing and integration, which is crucial for FL in multi-institutional settings. Standardization efforts are underway, but achieving seamless interoperability remains a long-term goal [113].

In online monitoring, variations in data collected from different types of wearable devices may minimize the accuracy of predictive models in healthcare applications [114].

### 6.4. Computational and Resource Constraints

Implementing FL in healthcare requires significant computational resources, particularly for processing complex healthcare data such as medical images. Some IoT devices and local systems in healthcare lack the computational power to handle resource-intensive model training, making it difficult to implement advanced AI techniques like DL in low-resource settings [40].

The need for high-performance hardware limits the deployment of FL in smaller healthcare facilities and remote areas, restricting access to advanced healthcare technologies in resource-constrained systems [115].

### 6.5. Regulatory and Ethical Challenges

The deployment of FL in healthcare also meets regulatory and ethical problems. Compliance with data protection laws like HIPAA and GDPR needs strict adherence to privacy standards, which can be challenging to maintain in a federated environment. Additionally, ethical concerns around data ownership, informed consent, and algorithmic transparency are growing as AI becomes more pervasive in healthcare [116].

## 7. Future Directions and Recommendations

As FL continues to progress, its potential in healthcare applications is increasingly evident. The decentralized nature of FL makes it well suited for privacy-sensitive industries like healthcare, where secure, collaborative data use is essential. To fully harness its benefits, however, several key areas require further research and development.

### 7.1. Enhancing Privacy and Security

Future research should prioritize developing more efficient privacy-preserving methods for FL. Techniques like secure multiparty computation, differential privacy, and homomorphic encryption can be improved to reduce computational overhead while maintaining robust data security [81]. Integrating blockchain with FL is also a promising direction; it could increase data traceability and integrity by creating an immutable ledger of updates, adding an extra layer of security [90].

### 7.2. Improving Scalability and Efficiency

As FL applications grow, optimizing scalability and efficiency will be essential. Research into lightweight FL algorithms that can operate effectively on low-power healthcare devices and IoT sensors is crucial. Techniques like Federated Averaging (FedAvg) and advanced data compression methods should be refined to minimize communication overhead, facilitating real-time data processing in resource-constrained settings [117].

### 7.3. Standardization and Interoperability

To foster widespread adoption, there is a need for standardized protocols and data formats that facilitate interoperability across healthcare institutions and devices. Developing universal standards for data sharing and model integration can improve collaboration among health organizations, especially in multi-institutional research initiatives [2]. This could also help address data heterogeneity issues, enhancing the generalizability and reliability of FL models.

### 7.4. Focus on Ethical and Regulatory Compliance

Future FL implementations must prioritize ethical considerations and regulatory compliance, especially given the sensitive nature of healthcare data. Transparent model interpretability techniques are needed to ensure that AI-driven decisions in healthcare are understandable and trustworthy. In parallel, collaboration with regulatory bodies to establish ethical frameworks and guidelines can streamline the integration of FL in healthcare, ensuring alignment with data protection laws like GDPR and HIPAA [69].

Ethical principles should prioritize transparency, compliance surveillance, privacy rights, and informed consent to guarantee the responsible use of FL in the medical field. This entails outlining goals and procedures precisely, obtaining continuous consent for data usage, giving stakeholders the authority to manage their data and conducting frequent audits to make sure ethical requirements and privacy regulations are being followed [118].

### 7.5. Encouraging Multidisciplinary Collaboration

Finally, federated learning in healthcare would benefit from multidisciplinary collaboration among data scientists, healthcare professionals, regulatory bodies, and technology developers. This collaborative approach can help address domain-specific challenges, refine FL algorithms for clinical relevance, and ensure that the technology aligns with practical healthcare needs [44].

Table 7 shows that AI in diagnostics and predictive analytics showcases diverse applications of AI, from enhancing diagnostic accuracy in medical imaging and pathology to predicting health events like cardiovascular risks, driving early interventions and personalized care. These advancements improve patient outcomes while streamlining healthcare workflows.

## 8. Conclusions

This review thoroughly investigated FL in smart healthcare systems, with a focus on how it might improve patient privacy and facilitate collaborative machine learning without sacrificing data security. This study demonstrates the capability of FL in real-time data pre-processing and predictive analysis in healthcare by highlighting its interaction with IoTs. Furthermore, we describe new privacy-preserving methods that are essential for getting around these restrictions while identifying important FL issues such data heterogeneity, adaptability, and security vulnerabilities.

This work contributes to clarifying how FL can help healthcare organizations use a variety of datasets while still complying with regulations, which can lead to better patient outcomes. Additionally, this research emphasizes how crucial it is to create uniform standards and procedures in order to improve FL systems’ compatibility across various healthcare environments.

Future directions for FL should focus on addressing scalability challenges and innovative approaches for lightweight FL algorithms, especially in IoT-constrained environments. In IoT-constrained situations, where gadgets frequently have restricted CPU resources, the development of lighter FL techniques is essential for scalability. Methods like quantization, model pruning, and federated distillation can drastically cut down on energy use and transmission overhead. Edge computing has the potential to improve instantaneously and guarantee scalability across a range of medical applications when paired with hybrid FL models that strike an equilibrium between physical and cloud-based processing.

## Figures and Tables

**Table 1:** Challenges and Opportunities in Federated Learning.

Category	Aspect	Description
**Challenges**	Data Privacy	Ensuring patient data privacy and compliance with regulations.
	Heterogeneous Device	Managing different computational capabilities across devices.
**Opportunities**	Collaborative Research	Enabling research collaboration across geographical boundaries.
	Blockchain Integration	Improving data security using blockchain with FL.

**Table 2:** Comparison with existing review articles on FL in smart healthcare.

Ref	Focus	Privacy and Security	Challenges/ Limitations	Real-World Implementations	Unique Contributions
[29]	FL in IoMT applications	Data fragmentation, and privacy concerns	Scalability issues, lack of sophisticated privacy methods	Limited real-world case studies	Preliminary investigation of IoMT privacy issues
[30]	FL in smart healthcare	Differential privacy strategies	Data exchange, ethical dilemmas, and inadequate security risk assessment	General healthcare case studies	Thorough analysis of FL in the medical field
[31]	FL in smart cities	Decentralized training algorithms	Security risks, data tampering, and interoperability between systems, minimal healthcare-specific focus	Blockchain integration conceptualization, and edge computing	Broad viewpoint on FL applications
[32]	Taxonomy of FL in medical applications	Fundamental data privacy issues	Data aggregation challenges, lack of comprehensive privacy solutions	No specific real-world implementations discussed	Systematic classification of FL in the medical field
[33]	FL techniques in healthcare informatics	Basic encryption techniques	Potential threats of adversarial attacks, lack of useful implementation guaidance	Suggested applications but not specified	Detailed technique review
[34]	FL in healthcare	Privacy-protecting approaches	Minimal attention on IoT integration, lacks in-depth analysis of adaptability in IoT healthcare	Case studies focused on healthcare but not focusing IoTs	FL applications in healthcare focusing conventional clinical data without IoTs
**Ours**	FL’s integration with IoMT devices, wearables, and remote monitoring systems for predictive analytics and personalized care	Advanced privacy-preserving techniques such as differential privacy, secure multiparty computation, and adversarial threat detection	Scalability issues, model inversion, and adversarial attacks; offers practical advice for further study	Detailed real-world case studies included with IoT integration	Thorough examination of privacy-preserving strategies in healthcare environments with IoT constraints

**Table 3:** Sample data extraction.

Focused Work	Authors	Year	Application Area	Contribution	Challenges	Proposed Solutions
FL in Disease	[35]	2024	Disease Prediction	New FL model	Data Quality	Federated Averaging
Privacy Enhancement in FL	[36]	2024	Privacy Techniques	Privacy Techniques for Data	Data Privacy	Differential Privacy
Challenges in FL for Health care	[37]	2024	Challenges in FL	Discussion on Challenges	Model Robustness	Robust Algorithms
Future of FL in Healthcare	[38]	2024	HER analysis	Proposed future direction	Scalability	Scalable FL Framework

**Table 4:** Federated learning in healthcare: applications, improvements over centralized servers, and limitations.

Application	FL Improvement over Centralized Models	Critical Evaluation of Limitations/Effectiveness/Impact	Ref
**Disease Prediction**	FL enables secure, decentralized training on patient data across hospitals, preserving privacy and minimizing data breach risks.	Limited cross-institutional collaboration may bias models, while data heterogeneity can hinder performance. Real-world validation is still needed to ensure effectiveness.	[50]
**Remote Monitoring**	FL processes wearable device data without sharing personal health information, enabling continuous learning across devices.	Inconsistent device data quality and intermittent availability pose challenges to model accuracy and training.	[51]
**Medical Image Diagnosis**	FL improves model performance by leveraging diverse, distributed medical image datasets without centralizing storage.	Data imbalance and communication overhead hinder FL’s generalization and real-time use.	[27]
**Personalized Treatment Plans**	FL shares knowledge from diverse patient populations while protecting patient data, enabling more accurate, personalized treatments.	Variations in local datasets complicate combining treatment protocols, while high computational costs may limit scalability.	[52]
**Clinical Trial Recruitment**	FL helps identify trial candidates across institutions, improving recruitment strategies while protecting sensitive information.	Inconsistent inclusion/exclusion criteria and lack of central oversight may reduce recruitment accuracy.	[32]
**Chronic Disease Management**	FL aggregates data from chronic patients, enhancing predictive models for disease progression and management.	Lack of data from underrepresented groups can bias predictions, reducing fairness and accuracy.	[41]
**Real-Time Alerting Systems**	FL processes real-time data at the edge (e.g., wearables), improving patient response times and reducing latency	Data quality and processing constraints can delay or skew alerts, while intermittent data or device malfunctions affect reliability.	[53]
**Drug Discovery**	FL enables pharmaceutical companies to share research data while protecting intellectual property, accelerating drug discovery.	Protocol and data format variations hinder model training, and FL needs improvement in handling diverse data types.	[54]
**Predictive Health Analytics**	FL enables predictive models to learn from diverse data sources (hospital records, wearables) while preserving patient privacy.	Inconsistent data can lead to inaccurate predictions, and FL models may struggle with rare diseases due to limited data.	[33]
**Patient Privacy and Security**	FL preserves patient data privacy by localizing it, enabling collaborative learning without unauthorized access to sensitive data.	FL improves privacy, but communication protocols may remain vulnerable to attacks, and ensuring patient consent and transparency is challenging.	[55]

**Table 5:** Summary of threats and mitigation techniques.

Ref	Threat	Description	Mitigation Techniques	Methodology
[69]	Gradient Leakage	Gradients encode sensitive information, allowing attackers to reconstruct input data.	Noise Addition (Differential Privacy)	Use of Differential Privacy in Training
[81]	Additional Leakage	Malicious participants manipulate aggregation to bias the global model	Secure Aggregation (Homomorphic Encryption)	Homomorphic Encryption for Secure Aggregation
[2]	Participant Data Leakage	Unauthorized access to participant updates leads to privacy breaches	Encryption of Communications and Data	End-to-End Encryption for Data Transmission
[32]	Sybil Attacks	Attackers create multiple fake identities to compromise the FL process	Identity Verification (e.g., Blockchain)	Blockchain for Identity Management
[84]	Data Poisoning	Attackers create multiple fake identities to compromise the FL process	Data Validation to Ensure Authenticity	Authentication and Validation in Data Collection
[44]	Backdoor Insertion	Attackers embed hidden functionalities or vulnerabilities in the model	Model Validation to Detect Backdoors	Advanced Model Validation Techniques
[33]	Model Update Eavesdropping	Interception of updates in transit can expose sensitive data	Secure Communication Protocols (e.g., TLS)	Secure Transmission via TLS
[85]	Byzantine Faults	Faulty or malicious participants send incorrect updates to disrupt training	Fault Tolerance Mechanisms	Incorporation of Fault Tolerance Algorithms
[86]	Inference Attacks	Sensitive details about training data are inferred from the global model	Differential Privacy to Add Output Noise	Adding Output Noise to Protect Inferences
[50]	Update Injection	Unauthorized updates are injected to manipulate the global model	Update Screening to Detect Suspicious Changes	Screening and Monitoring of Model Updates

**Table 6:** Summary of features and descriptions showing how FL preserves patient privacy across institutions.

Ref	Features	Description
[2]	Data Privacy	Protect sensitive patient data while enabling collaboration
[105]	Regulatory Compliance	Meets HIPAA and GDPR standards
[69]	Improved Model Robustness	Leverages diverse datasets for more accurate predictions
[106]	Case Study	Uses a COVID-19-predictive model across European hospitals

**Table 7:** AI in diagnostics and predictive analytics.

Ref	Application	Example Use Case	Advantages
[119]	Cardiology	AI applications predicting cardiovascular events using electronic health records	Facilitate preventive measures, reducing the incidence of heart attacks and strokes
[120]	Medical Imaging	AI algorithms detecting breast cancer in mammograms	Increase diagnostic accuracy, reduces false positives, and expedites treatment
[121]	Pathology	AI systems analyzing histopathological slides for tumor classification	Enhance diagnostic precision and consistency across pathologists
[122]	Genomic Analysis	AI-driven interpretation of genomic data for personalized cancer therapies	Identifies actionable mutations, tailoring treatments to individual patients
[123]	Ophthalmology	AI systems detecting diabetic retinopathy in retinal images	Enable early treatment, preventing vision loss in diabetic patients
[124]	Radiology Workflow	AI tools prioritizing radiology worklists based on urgency	Improve efficiency, ensuring critical cases receive prompt attention
